# CD8^+^-T Cells With Specificity for a Model Antigen in Cardiomyocytes Can Become Activated After Transverse Aortic Constriction but Do Not Accelerate Progression to Heart Failure

**DOI:** 10.3389/fimmu.2018.02665

**Published:** 2018-11-15

**Authors:** Carina Gröschel, André Sasse, Sebastian Monecke, Charlotte Röhrborn, Leslie Elsner, Michael Didié, Verena Reupke, Gertrude Bunt, Andrew H. Lichtman, Karl Toischer, Wolfram-Hubertus Zimmermann, Gerd Hasenfuß, Ralf Dressel

**Affiliations:** ^1^Institute of Cellular and Molecular Immunology, University Medical Center Göttingen, Göttingen, Germany; ^2^DZHK (German Center for Cardiovascular Research), Partner Site Göttingen, Göttingen, Germany; ^3^Institute of Pharmacology and Toxicology, University Medical Center Göttingen, Göttingen, Germany; ^4^Department of Cardiology and Pneumology, University Medical Center Göttingen, Göttingen, Germany; ^5^Central Animal Facility, University Medical Center Göttingen, Göttingen, Germany; ^6^Clinical Optical Microscopy, Department of Neuropathology, University Medical Center Göttingen, Göttingen, Germany; ^7^Department of Pathology, Brigham and Women's Hospital, Harvard Medical School, Boston, MA, United States

**Keywords:** autoimmunity, autoantigen, pressure overload, cytotoxic T cells, adoptive transfer, transgenic T cell receptor, heart failure

## Abstract

Heart failure due to pressure overload is frequently associated with inflammation. In addition to inflammatory responses of the innate immune system, autoimmune reactions of the adaptive immune system appear to be triggered in subgroups of patients with heart failure as demonstrated by the presence of autoantibodies against myocardial antigens. Moreover, T cell-deficient and T cell-depleted mice have been reported to be protected from heart failure induced by transverse aortic constriction (TAC) and we have shown recently that CD4^+^-helper T cells with specificity for an antigen in cardiomyocytes accelerate TAC-induced heart failure. In this study, we set out to investigate the potential contribution of CD8^+^-cytotoxic T cells with specificity to a model antigen (ovalbumin, OVA) in cardiomyocytes to pressure overload-induced heart failure. In 78% of cMy-mOVA mice with cardiomyocyte-specific OVA expression, a low-grade OVA-specific cellular cytotoxicity was detected after TAC. Adoptive transfer of OVA-specific CD8^+^-T cells from T cell receptor transgenic OT-I mice before TAC did not increase the risk of OVA-specific autoimmunity in cMy-mOVA mice. After TAC, again 78% of the mice displayed an OVA-specific cytotoxicity with on average only a three-fold higher killing of OVA-expressing target cells. More CD8^+^ cells were present after TAC in the myocardium of cMy-mOVA mice with OT-I T cells (on average 17.5/mm^2^) than in mice that did not receive OVA-specific CD8^+^-T cells (3.6/mm^2^). However, the extent of fibrosis was similar in both groups. Functionally, as determined by echocardiography, the adoptive transfer of OVA-specific CD8^+^-T cells did not significantly accelerate the progression from hypertrophy to heart failure in cMy-mOVA mice. These findings argue therefore against a major impact of cytotoxic T cells with specificity for autoantigens of cardiomyocytes in pressure overload-induced heart failure.

## Introduction

In many clinical studies signs of inflammation have been observed during the progression of chronic heart failure (1). Inflammation is thereby not restricted to heart failure occurring in the cause of classic inflammatory cardiomyopathies. It can occur also in response to hemodynamic overload (2). Increased levels of pro-inflammatory cytokines including interleukin (IL)-6 and tumor necrosis factor (TNF)-α were observed in the circulation of patients with pressure overload (3, 4). However, clinical studies targeting TNF-α by anti-inflammatory drugs have been largely unsuccessful in the therapy of heart failure (5, 6). Therefore, the pathophysiological basis of the inflammatory response to hemodynamic load needs to be further investigated to identify better therapeutic targets.

In addition to the unspecific immune response to tissue damage or cellular stress that is mediated by cells of the innate immune system, autoimmune responses of the adaptive immune system to myocardial antigens can contribute to the progression of heart failure. This is most obvious from studies of autoantibodies against myocardial antigens. Antibodies, e.g., against β1-adreno-receptors and M2-acetylcholine-receptors were found after transverse aortic constriction (TAC) in animal models (7). In patients, such autoantibodies of the IgG isotype can directly impair cardiac function (8, 9) and are therefore promising therapeutic targets (10, 11). Autoreactive T helper cells must also exist in these patients, since their help is required for immunoglobulin class switching to IgG in the autoreactive B cells.

We have recently demonstrated that T helper cells with specificity for an antigen in cardiomyocytes can contribute to the progression of heart failure after TAC also independently of autoantibodies (12). For this purpose, cMy-mOVA mice expressing the model antigen ovalbumin (OVA) on cardiomyocytes (13) were crossed with OT-II mice (14) expressing a transgenic T cell receptor (TCR) on CD4^+^-T cells with specificity for OVA. In the resulting double-transgenic cMy-mOVA-OT-II mice, progression from hypertrophy to heart failure after TAC was accelerated compared to cMy-mOVA mice. No OVA-specific antibodies were found after TAC but more T cells infiltrated the myocardium of cMy-mOVA-OT-II than cMy-mOVA mice where they could directly contribute to maladaptive cardiac remodeling (12).

It has not been investigated so far, whether CD8^+^-cytotoxic T lymphocytes (CTL) with specificity for an antigen in cardiomyocytes become also activated in response to pressure overload and contribute to the progression of heart failure. To address these questions, we analyzed whether CD8^+^-T cells with specificity against OVA become activated in cMy-mOVA mice after TAC. Moreover, we adoptively transferred CD8^+^-T cells with specificity for OVA from OT-I mice, carrying a transgenic TCR with specificity for OVA on CD8^+^-T cells (15), into cMy-mOVA mice before TAC to investigate whether they would contribute to the progression of heart failure. We show here that CTL with specificity for an antigen in cardiomyocytes indeed can become activated after TAC but fail to accelerate progression into heart failure.

## Materials and methods

### Animal experiments

All animal experiments were approved by the responsible agency (Niedersächsisches Landesamt für Lebensmittelsicherheit und Verbraucherschutz) and were carried out in compliance with German and European legislation (Directive 2010/63/EU). OVA-transgenic cMy-mOVA (13), TCR-transgenic OT-I (15), and double-transgenic cMy-mOVA-OT-I mice were bred in the central animal facility at the University Medical Center Göttingen under specific pathogen-free conditions in individually ventilated cages and in a 12 h light-dark cycle. Both, OT-I and cMy-mOVA mice have a C57BL/6 background. Mice aged between 8 and 12 weeks were used for experiments. Male and female mice were equally distributed but otherwise randomly assigned to the experimental groups. The cMy-mOVA mice that received OVA-specific CD8^+^-T cells purified by magnetic activated cell sorting (MACS) from lymph nodes of OT-I mice are designated cMy-mOVA+OT-I mice. The adoptive transfer of 10^7^ CD8^+^-T cells was performed by intravenous injection into the tail vein 1 day before surgery.

TAC and sham surgery was performed as described previously (12, 16). Briefly, the mice received intraperitoneal injections of medetomidin (0.5 mg/kg), midazolam (5 mg/kg), and fentanyl (0.05 mg/kg) for anesthesia. The transversal aorta was displayed after horizontal incision at the jugulum and a 26-gauge needle was tied against the aorta. The surgical thread was not tied in sham-operated mice. The skin was closed after removal of the needle and the anesthesia was reversed by subcutaneous injection of atipamezol (2.5 mg/kg) and flumazenil (0.5 mg/kg). The mice received buprenorphine (60 μg/kg) subcutaneously 1 h after surgery for further analgesia. Metamizole (1.33 mg/ml) had to be added to the drinking water for 1 week to achieve long-term analgesia.

The pressure gradient over the aortic ligature was measured using pulsed wave Doppler 3 days after surgery. At this time point, approximately 50 μl blood were taken from the orbital sinus of the mice to determine the presence of adoptively transferred CD8^+^-T cells in the blood of cMy-mOVA+OT-I mice. For echocardiography, the mice were anesthetized with 3-% isoflurane, and temperature, respiration, and ECG-controlled anesthesia was maintained with 1.5-% isoflurane.

At the end of the experiments, 10 weeks after the operation, the mice were sacrificed in isoflurane anesthesia by cervical dislocation. The hearts were excised, perfused via the aorta with 0.9% NaCl and after weighting of the ventricles, one-third of the heart from the middle part was fixed in 3.7% formaldehyde solution overnight and the other two thirds oriented toward the basis and the apex of the heart were snap frozen in liquid nitrogen. Finally, spleens were harvested and placed in Dulbecco's modified Eagle medium (DMEM) on ice for further analysis.

### Echocardiography

A Vevo2100 (VisualSonics, Toronto, Canada) system with a 30 MHz center frequency transducer was used for transthoracic echocardiography. B-mode recordings (16, 17) were used to determine the long axis in systole (Ls) and diastole (Ld), the end-diastolic (LVIDd) and end-systolic (LVIDs) left ventricular (LV) chamber diameter and the anterior and posterior wall thickness in systole (AWThs and PWThs) and diastole (AWThd and PWThd), the area of the endocardium in systole (Area s) and diastole (Area d) and the area of the epicardium in systole (Epi s). The recordings and analyses were done blinded to the treatment of the mice. Fractional area shortening (FAS) was calculated as (Area d–Area s)/Area d × 100. The ejection fraction (EF) was calculated as (5/6 × Area d × Ld−5/6 × Area s × Ls)/(5/6 × Area d × Ld) × 100. Echocardiographic LV weight (LVW) was estimated using the formula: 1.05 × 5/6 x (Epi s × (Ls + (AWThs + PWThs)/2)–Area s × Ls).

### Histology and immunohistochemistry

Formaldehyde-fixed heart samples were embedded in paraffin before 5 μm sections were cut. Collagen was visualized by Sirius Red staining to measure the extent of fibrosis as described previously (12, 17). Presence of immune cells in the myocardium was determined by immunohistochemistry (12, 18) using anti-CD3 (1:200, MCA1477, rat IgG_1_, ABD Serotec, Oxford, UK), anti-CD4 (1:200, clone 4SM95, rat IgG_1_, eBiosciences, Frankfurt, Germany), anti-CD8 (1:200, clone 4SM15, rat IgG_2a_, eBiosciences), anti-CD45R(B220) (1:200, clone RA3-6B2, rat IgG_2a_, Biolegend, Fell, Germany), and anti-F4/80 monoclonal antibodies (1:200, clone A3-1, rat IgG_2b_, Biolegend), respectively. For all antibodies except anti-F4/80, antigen retrieval was performed by boiling the slides 5 times for 5 min in sodium citrate buffer (10 mmol/L sodium citrate, pH 6, 0.05% Tween 20). Polyclonal biotinylated goat anti-rat IgG secondary antibodies (1:200, 112-065-062, Jackson laboratories) and HRP-conjugated streptavidin (405210, Biolegend) served as secondary and tertiary reagents.

The slides were scanned with a 20x objective (UPlanApo, NA 0.75) using the dotSlide SL slide scanner (Olympus, Hamburg, Germany) equipped with a peltier-cooled XC10 camera. The extent of fibrosis and the numbers of stained cells in two complete heart sections were quantified using cellSens Dimensions software (Olympus) by a scientist blinded to the treatment of the mice. Fibrotic areas were determined as the proportion of the area of collagen relative to the sum of the area of collagen and the area of cardiomyocytes.

### Lymphocyte preparation

Lymphocytes were obtained from lymph nodes of OT-I mice for adoptive transfers and from spleens of the cMy-mOVA and cMy-mOVA+OT-I mice in the experiments for analysis using a Tenbroeck homogenizer. Untouched CD3^+^CD8^+^ cells from OT-I mice were obtained by MACS (130-104-075, Miltenyi Biotec GmbH, Bergisch Gladbach, Germany) according to the manufacturer's protocol. The CD8^+^-T cells were incubated for 5 min with 5 μM of the dye carboxyfluorescein succinimidyl ester (CFSE; C-1157, Invitrogen) in phosphate buffered saline (PBS)/0.1% bovine serum albumin at 37°C and washed 3 times with DMEM containing 10% fetal calf serum (FCS) before adoptive transfer. The splenocytes of experimental animals were subjected to a removal of erythrocytes by incubation for 5 min in lysis buffer (155 mM NH_4_Cl, 10 mM KHCO_3_, 0.1 mM EDTA, pH 7.4-7.8).

### Flow cytometry

Flow cytometry was performed as described previously (19) on a FACS Calibur flow cytometer (BD Biosciences, Heidelberg, Germany) using CellQuestPro data acquisition and analysis software. Antibodies used for flow cytometry (anti-CD3, clone 17A2, rat IgG_2b_, PE-labeled; anti-CD8, clone 53-6.7, rat IgG_2a_ PE/Cy5-labeled; anti-TCRVβ5.1/5.2, clone MR9-4, mouse IgG_1_, FITC-labeled) and the respective isotype controls were purchased from Biolegend. The anti-H2K^b^/SIINFEKL antibody (clone 25-D1.16, mouse IgG_1_, APC-labeled) was obtained from BD Biosciences. For staining of cell surface antigens, 5 × 10^5^ cells were incubated in 100 μl PBS with 1 μg of the respective primary monoclonal antibody for 30 min at 4°C before washing with PBS. Blood samples were directly stained and processed with BD FACS Lysing Solution (#349202, Becton Dickinson) according to the manufacturer's instructions.

### Target cells to determine OVA-specific cytotoxicity

To monitor OVA-specific cytotoxicity, an enhanced green fluorescent protein (eGFP) or OVA-eGFP fusion protein expression cassette under control of an ubiquitously active hEF1α/CAG composite promoter in the pEGFP-1 vector (Clontech, Heidelberg, Germany) was introduced into the mouse leukemia cell line RMA (20), which carries the major histocompatibility complex (MHC) haplotype (H2^b^). For transfection, 10^7^ cells were mixed with 40 μg of the linearized vector before electroporation (250 mV and 960 μF). Successfully transfected cells were selected with 1,000 ng/ml G418 for 2 weeks before single cell clones were picked. Expression of eGFP was determined by flow cytometry to select stably transfected clones. The characterization of the selected clones expressing eGFP (RMA-con) or the OVA-eGFP fusion protein (RMA-OVA) is shown in Supplementary Figure 1.

### Cytotoxicity assay

The cytotoxic effector cells were used either directly at the day of preparation (day 0) or after restimulation with OVA for 4 days in ^51^Cr-release assays. For restimulation, the splenocytes were cultured in round-bottomed microtiter plates (5 × 10^5^ cells per well) in DMEM with 1 μM OVA and 10 ng/ml mouse IL-2 (#12340026, Immunotools, Friesoythe, Germany). Target cells were labeled by incubating 1 x 10^6^ cells in 200 μl DMEM containing 100 μl FCS and 50 μCi ${\text{Na}}_{2}^{51}$ CrO_4_ (Hartmann Analytic, Braunschweig, Germany) for 1 h at 37°C and washed three times with DMEM. Effector cells were added to 5 x 10^3^ ^51^Cr-labeled target cells in triplicates at various effector to target (E:T) ratios in 200 μl DMEM with 10% FCS per well of round-bottomed microtiter plates. The E:T ratios always indicate the ratio of CD3^+^CD8^+^ effector cells to target cells. Spontaneous release was determined by incubation of target cells in the absence of effector cells. The microtiter plates were centrifuged for 5 min at 40x g, incubated at 37°C for 4 h, and then centrifuged again. Supernatant and sediment were separately taken to determine radioactivity in each well using a MicroBeta^2^ counter (PerkinElmer Life Sciences, Köln, Germany). Percentage of specific lysis was calculated by subtracting percent spontaneous ^51^Cr-release (20). The resistance of parental RMA cells and the transfected clones to killing by MACS-separated IL-2-activated natural killer (NK) cells was determined by ^51^Cr-release assays in comparison to YAC-1 target cells as described previously (21).

### Statistics

Results are shown as means with standard error of the mean (SEM). The data were evaluated with the SPSS software (IBM, Armonk, NY, USA). Analyses of variance (ANOVA) was used to compare data sets with more than two experimental groups and the Bonferroni *post hoc* test was employed for subsequent comparisons between the groups. Cytotoxicity data were analyzed by 2-way ANOVA adjusted for E:T ratios. Mixed linear models with the specification auto-regressive process AR (1) were employed to analyze alterations over time in the echocardiography data sets. Data of two groups such as sham and TAC were compared by *t*-test. If the Levene test indicated inequality of variances, an unequal variance *t*-test has been used instead of Student's *t*-test. The Kolmogorov-Smirnov test was used to assess normal distribution. More than two groups of not normally distributed data were analyzed by the Kruskal-Wallis test. The Mann-Whitney *U*-test was used to compare two groups of not normally distributed data. The Kruskal-Wallis test and the *U*-test were also used frequently when only some data within a related set of data were not normally distributed in order to allow for uniform reporting of the analyses. The Bonferroni-Holm correction was used to adjust for multiple testing in *post hoc* comparisons of two groups. Categorical data were analyzed by Fisher‘s exact test. The survival curves of mice were compared by Log Rank (Cox-Mantel) tests. *P*-values of < 0.05 in two-sided tests were considered to be significant and three levels of significance are usually indicated in the figures (^*^P < 0.05, ^**^P < 0.01, ^***^P < 0.001).

## Results

### OVA-specific CTL can become activated in cMy-mOVA mice after TAC

**Figure 1:**
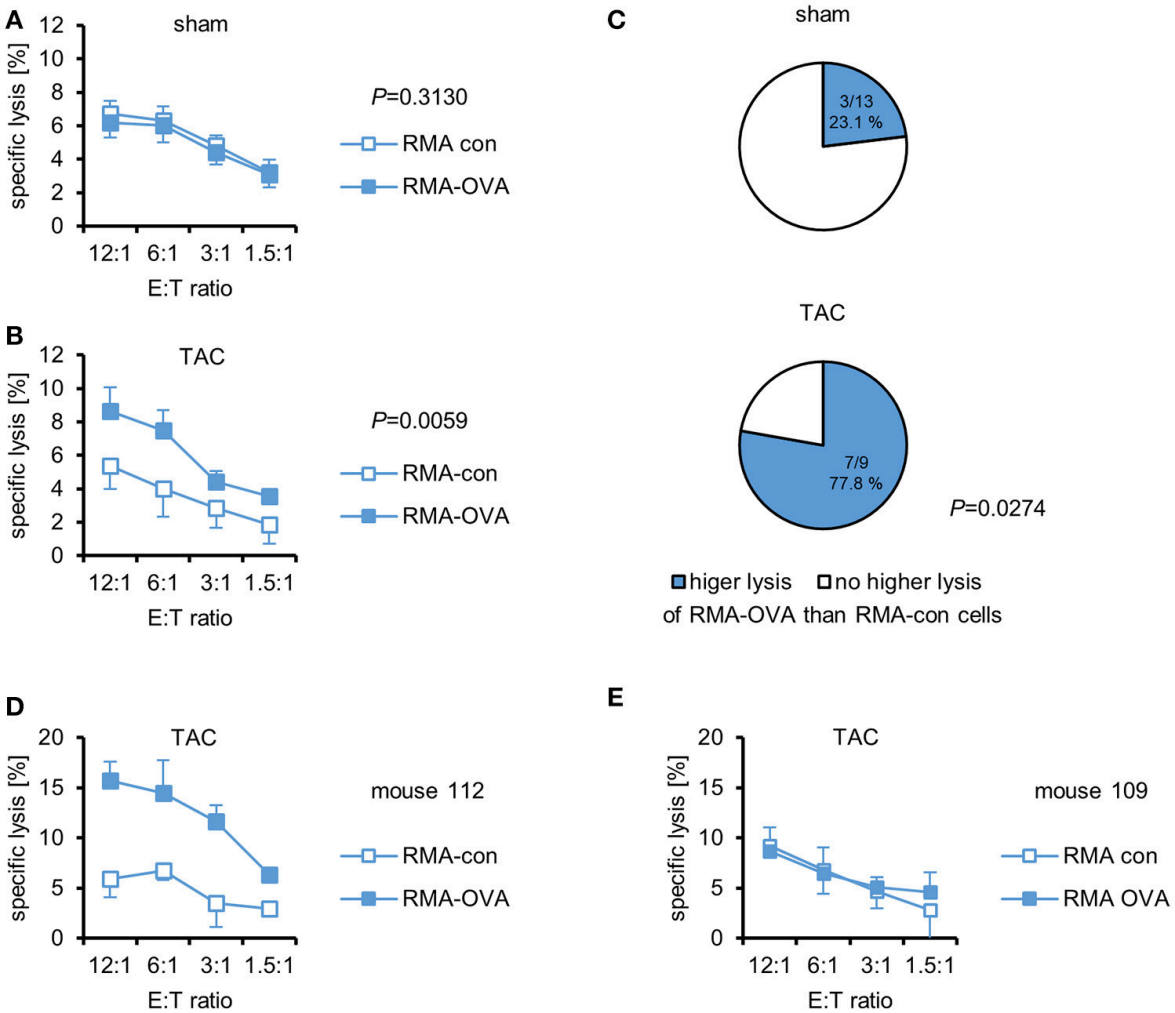
OVA-specific CTL become activated in cMy-mOVA mice after TAC. The cytotoxic activity of splenocytes against RMA-OVA and RMA-con target cells was determined by ^51^Cr-release assays at several CD3^+^CD8^+^ effector cell to target cell (E:T) ratios 10 weeks after **(A)** sham (*n* = 13) or **(B)** TAC (*n* = 9) surgery. Means and standard errors of the mean (SEM) are displayed. Differences between sham and TAC-operated mice were analyzed by 2-way ANOVA adjusted for the E:T ratios and the respective *P*-values are indicated. **(C)** The proportion of mice exerting a higher cytotoxic activity against RMA-OVA than RMA-con cells is displayed. The difference between sham and TAC-operated mice has been analyzed by Fisher‘s exact test and the *P*-value is displayed. **(D)** The OVA-specific cellular cytotoxicity was higher in some mice after TAC. Means and SEM of triplets are shown for mouse 112. **(E)** The lack of an OVA-specific cytotoxicity in mouse 109 that also underwent TAC is shown for comparison.

The investigation of the potential role of CTL in cardiac autoimmunity elicited by pressure overload is hampered by the lack of known relevant autoantigens. Therefore, we used cMy-mOVA mice that express OVA on the plasma membrane of cardiomyocytes (13) to determine whether a CTL response to this model antigen occurs after TAC. Splenocytes were harvested 10 weeks after TAC or sham surgery and re-stimulated *in vitro* for 4 days with 1 μM OVA. Afterwards, the cells were used as effector cells in ^51^Cr release assays against mouse leukemia RMA cells, which express either an OVA-EGFP fusion protein (RMA-OVA), and are therefore targets for OVA-specific CTL, or EGFP only as control (RMA-con). The characteristics of these target cell lines that were generated to measure OVA-specific CTL responses are shown in Supplementary Figure 1. Both, RMA-con and RMA-OVA cells were hardly killed by splenocytes from sham-operated mice (Figure 1A). Splenocytes from TAC-operated mice, in contrast, killed RMA-OVA cells significantly better than RMA-con cells (Figure 1B). The presence of an OVA-specific cellular cytotoxic activity against RMA target cells, which are resistant against NK cells, demonstrates that indeed OVA-specific CTL became activated in response to cardiac pressure overload although the specific lysis of OVA-expressing target cells was on average still low. In accord with these data, a significantly higher proportion of mice that underwent TAC (77.8%) than sham surgery (23.1%) exerted a higher cytotoxic activity against RMA-OVA than RMA-con cells (*P* = 0.0274, Fisher's exact test; Figure 1C). Notably, in some mice, the OVA-specific cytotoxicity after TAC was considerably higher than on average (Figure 1D), whereas in others no OVA-specific CTL activity was detected (Figure 1E).

### OVA-specific CD8^+^-T cells do not accelerate progression of cMy-mOVA mice into heart failure

The observation of a cytotoxic activity against a myocardial antigen after TAC justified further investigation. To increase the likelihood and potentially also the strength of an OVA-specific CTL response after TAC, we crossed cMy-mOVA mice with OT-I mice to obtain animals that express OVA on cardiomyocytes and have CD8^+^-T cells mostly with specificity for this antigen. Previously, we have used this strategy successfully to generate cMy-mOVA-OT-II mice that have CD4^+^-T cells with specificity for OVA (12). Unexpectedly, all of these double-transgenic cMy-mOVA-OT-I mice died before reaching an age of 10 days (Supplementary Figure 2A). Their myocardium did not display an infiltration of CD3^+^-T cells (Supplementary Figure 2B) making it unlikely that a vigorous autoimmune response of OVA-specific CTL to OVA-expressing cardiomyocytes was the underlying reason of death. However, we could not continue to bread these mice to determine the actual reason of death and decided to circumvent this problem by adoptively transferring OVA-specific CD8^+^-T cells from OT-I mice into cMy-mOVA mice before surgery.

Thus, cMy-mOVA mice received at day 1 before TAC or sham surgery 10^7^ MACS-separated CFSE-labeled CD3^+^CD8^+^ cells from OT-I mice (cMy-mOVA+OT-I) or PBS (cMy-mOVA) by intravenous injection (*n* = 14 per group). The transgenic TCR of OT-I mice was detected by a TCRVβ5.1/5.2-specific antibody. The purity of the transferred CD8^+^-T cells carrying the transgenic Vβ5.1/5.2^+^ TCR was >90% (Supplementary Figure 3). Three days after surgery, the pressure gradient over the aortic ligature was determined using pulsed wave Doppler. In all mice that underwent TAC an aortic stenosis was obtained. The pressure gradients in mice that had received CD8^+^-T cells from OT-I mice before TAC was not significantly different from control mice after TAC (Supplementary Figure 4A). At day 3 after surgery, also blood was taken to verify by flow cytometry the successful transfer of OT-I-derived cells. On average about 2% of the lymphocytes in the blood were CD3^+^CFSE^+^ OT-I-derived T cells (Supplementary Figure 4B) suggesting that most transferred cells were at this time point in other compartments than the peripheral blood. Signs of a dilution of CFSE due to cell proliferation were not observed in the non-lymphocyte-depleted recipients. Six mice (3 after sham and 3 after TAC intervention) were at this point excluded from further analysis since <0.5% of the lymphocytes in the blood of the recipients were CD3^+^CFSE^+^ T cells. A few additional mice (sham: *n* = 3, TAC: *n* = 4) were operated and already sacrificed at the end of the first week in order to exclude an early loss of the transferred T cells. Similar proportions (on average 2.3%) of CFSE-labeled T cells were found among the splenocytes of sham and TAC-operated mice (Supplementary Figure 4C). Furthermore, the proportion of TCRVβ5.1/5.2^+^-T cells was increased in these mice compared to cMy-mOVA mice (*n* = 7) that did not receive OT-I-derived T cells (Supplementary Figure 4D), indicating that OVA-specific T cells were still present in both sham and TAC-operated mice 1 week after the intervention.

**Figure 2:**
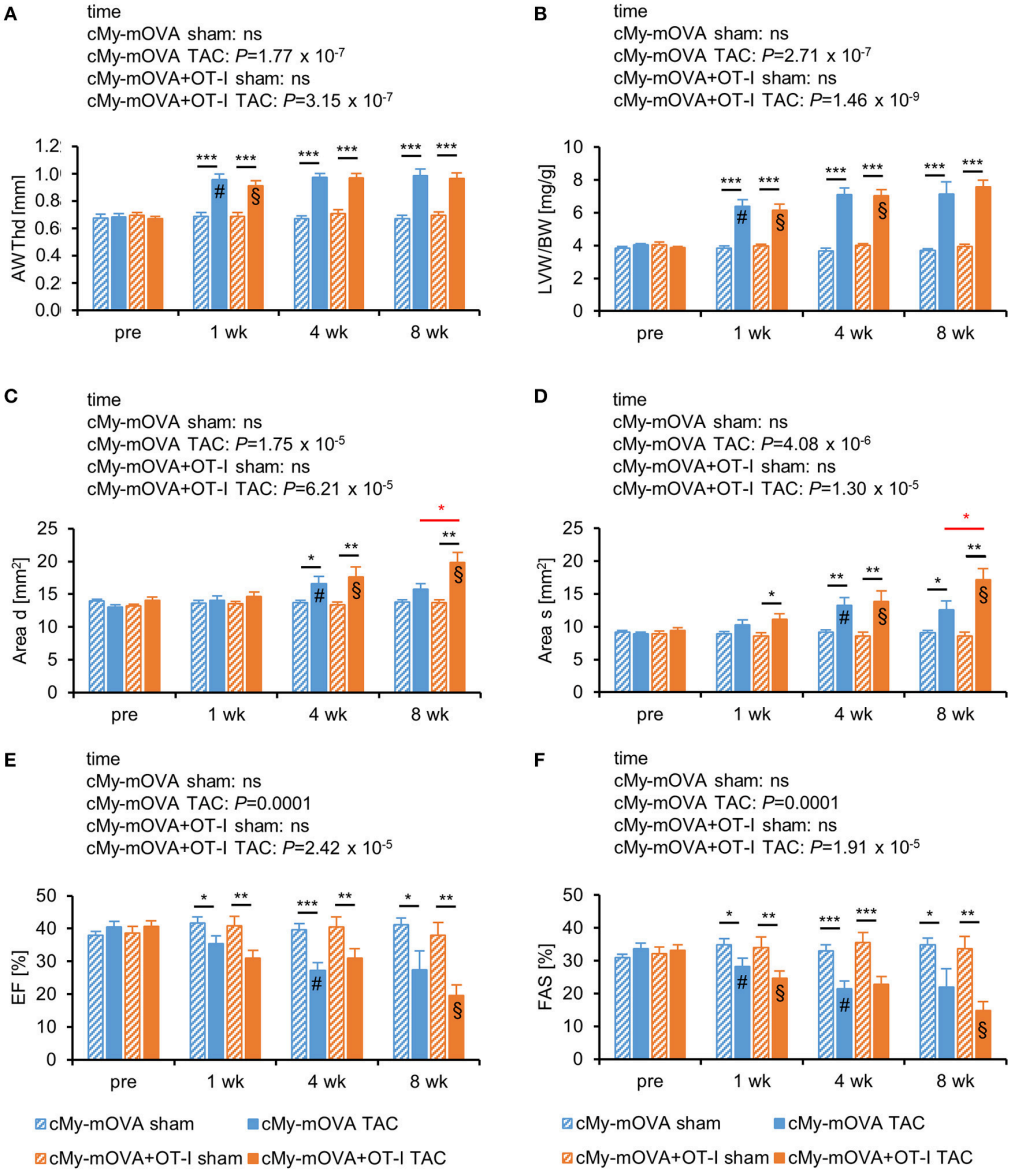
OVA-specific CD8^+^-T cells promote cardiac dilatation but do not accelerate the progression from hypertrophy to heart failure after TAC in cMy-mOVA mice. Echocardiography was performed in cMy-mOVA and in cMy-mOVA+OT-I mice before (pre; *n* = 14 per group for cMy-mOVA and *n* = 11 per group for cMy-mOVA+OT-I mice) as well as 1 week (*n* = 14 for cMy-mOVA and *n* = 11 for cMy-mOVA+OT-I mice), 4 weeks (*n* = 14 cMy-mOVA and *n* = 11 (sham) or 10 (TAC) for cMy-mOVA+OT-I mice), and 8 weeks (*n* = 14 (sham) or 10 (TAC) for cMy-mOVA and *n* = 11 (sham) or 9 (TAC) for cMy-mOVA+OT-I mice) after sham or TAC surgery. **(A)** Anterior wall thickness in diastole (AWThd), **(B)** left ventricular weight/body weight (LVW/BW) ratio, **(C)** area of the endocardium in diastole (Area d), **(D)** area of the endocardium in systole (Area s), **(E)**, ejection fraction (EF), and **(F)** fractional area shortening (FAS) were determined and means plus SEM are displayed. Differences between the time points within each experimental group were analyzed by a mixed linear model (time) and the *P*-values are given in the panels. Significant differences in the Bonferroni *post hoc* test (*P* < 0.05) compared to the previous time point are indicated for cMy-mOVA (#) and cMy-mOVA+OT-I mice (§). Differences between sham and TAC groups at a given time point were analyzed by *t*-tests and significant differences are indicated by black bars (^*^P < 0.05, ^**^P < 0.01, ^***^P < 0.001). Similarly, differences between cMy-mOVA and cMy-mOVA+OT-I mice after TAC were analyzed and red bars and stars indicate significant *P*-values.

Echocardiography was performed to determine heart function 3 days before and 1, 4, and 8 weeks after the intervention. Cardiac hypertrophy developed within 1 week after TAC as indicated by the anterior wall thickness in diastole (AWTHd; Figure 2A) and the left ventricular weight/body weight (LVW/BW) ratio (Figure 2B). A dilation of the left ventricle occurred in both experimental groups between the first and the fourth week as indicated by an increase of the area of the endocardium in diastole (Area d; Figure 2C) and the area of the endocardium in systole (Area s; Figure 2D). Notably, the dimensions of Area d and Area s further progressed from week 4 to week 8 only in cMy-mOVA+OT-I mice and both parameters were greater in the mice with OVA-specific CTL than in cMy-mOVA mice 8 weeks after TAC. However, heart function after TAC measured as ejection fraction (EF; Figure 2E) or fractional area shortening (FAS; Figure 2F) was not significantly different between cMy-mOVA+OT-I and cMy-mOVA mice at any time point. On average EF and FAS were, however, more reduced 10 weeks after TAC in cMy-mOVA+OT-I than cMy-mOVA mice. When the development of heart failure over time was analyzed for the individual groups, a significant reduction of the EF was observed in cMy-mOVA mice between week 1 and week 4 and in cMy-mOVA+OT-I mice between week 4 and week 8 (Figure 2E). The FAS was reduced in both cMy-mOVA+OT-I and cMy-mOVA mice already 1 week after TAC and declined further until week 4 in cMy-mOVA mice and from week 4 to week 8 in cMy-mOVA+OT-I mice. In summary, the presence of CD8^+^-T cells with specificity for an antigen in cardiomyocytes appeared to promote left ventricular dilation but failed to significantly accelerate the progression from hypertrophy to heart failure at least during the time period analyzed here.

All sham-operated mice survived until the end of the experiment and did not show any clinical signs of sickness during the course of the experiment. Four of the 14 cMy-mOVA mice died or had to be sacrificed before the end of the experiment due to illness (Supplementary Figure 5A). Of the 11 cMy-mOVA+OT-I mice, 9 remained in the experiment until the end of the observation time (Supplementary Figure 5B). The survival of the mice that had received OT-I-derived CD8^+^-T cells before TAC was not different from controls [*P* = 0.5608; Log Rank (Cox-Mantel) test], suggesting that the ventricular dilation observed in echocardiography does not translate into an enhanced heart failure related mortality.

### More T cells infiltrate the myocardium of cMy-mOVA+OT-I than cMy-mOVA mice, but fibrosis and cardiac hypertrophy are similar

**Figure 3:**
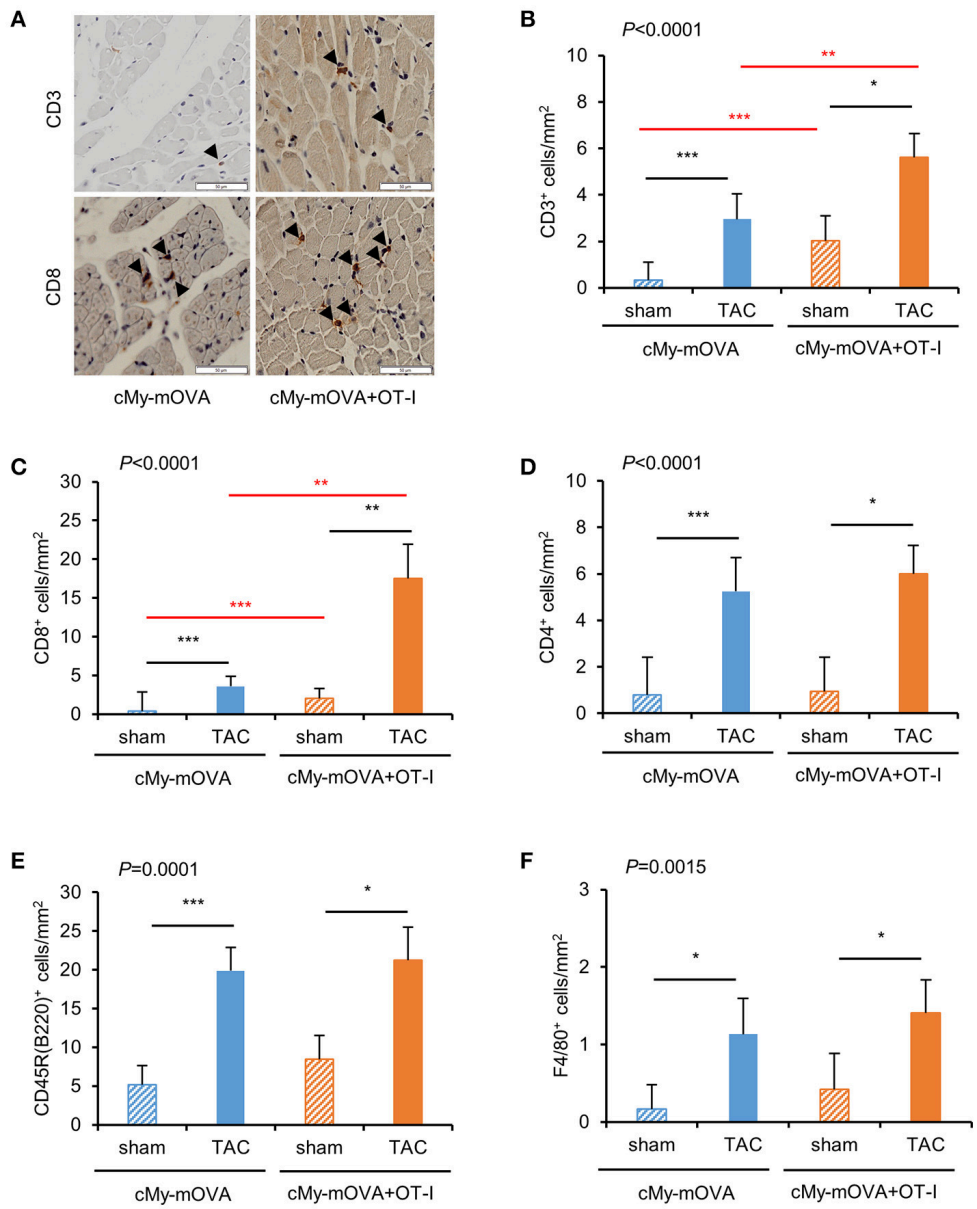
More infiltration of CD3^+^ and CD8^+^ cells in the myocardium of cMy-mOVA+OT-I than cMy-mOVA mice. **(A)** The infiltration of CD3^+^ and CD8^+^ cells was analyzed by immunohistochemistry in the myocardium of sham and TAC-operated cMy-mOVA and cMy-mOVA+OT-I as illustrated here. The bars indicate 50 μm. The arrow heads point to CD3^+^ or CD8^+^ cells, respectively. The numbers of infiltrating CD3^+^-T cells **(B)**, CD8^+^ cells **(C)** CD4^+^ cells **(D)**, CD45R(B220)^+^ B cells **(E)** and F4/80^+^ monocytes/macrophages **(F)** were determined in the myocardium after 10 weeks in sham and TAC-operated cMy-mOVA (sham *n* = 14, TAC *n* = 10) and cMy-mOVA+OT-I mice (sham *n* = 11, TAC *n* = 9) and means plus SEM are shown. The *P*-value of a Kruskal-Wallis test comparing all groups is indicated. Differences between two groups were analyzed by *U*-tests and significant *P*-values are given in the panels (^*^P < 0.05, ^**^P < 0.01, ^***^P < 0.001). The Bonferroni-Holm correction was used to adjust for multiple testing in the two group comparisons.

The mice were sacrificed 10 weeks after surgery. The infiltration of the myocardium with cells of the immune system was assessed by immunohistochemistry (Figure 3A). More CD3^+^-T cells were present in the myocardium of TAC than sham-operated mice (Figure 3B). Notably, more CD3^+^-T cells infiltrated the myocardium of cMy-mOVA+OT-I than cMy-mOVA mice after TAC but also after sham surgery. The same pattern was observed when the numbers of CD8^+^ cells were determined (Figure 3C), suggesting that adoptively transferred OT-I-derived CD8^+^-T cells were enriched in the myocardium of the cMy-mOVA+OT-I mice. CD4^+^ T cells (Figure 3D) and CD45R(B220)^+^ B cells (Figure 3E) as well as F4/80^+^ myeloid cells (Figure 3F), which include monocytes and macrophages, increased in numbers after TAC but were not more abundant in cMy-mOVA+OT-I than cMy-mOVA mice, arguing for a specific enrichment of the OVA-specific CD8^+^-T cells in the OVA-expressing myocardium.

**Figure 4:**
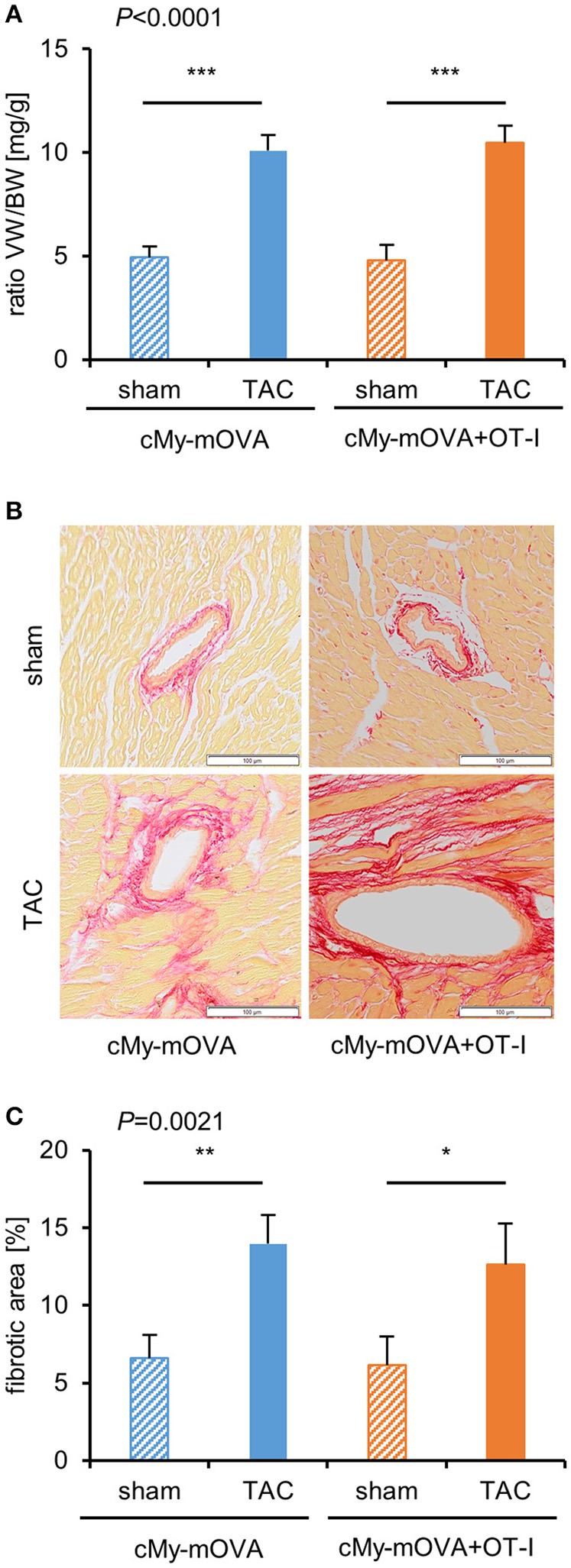
Similar cardiac hypertrophy and fibrosis in cMy-mOVA and cMy-mOVA+OT-I mice after TAC. **(A)** The ventricular weight/body weight (VW/BW) ratio was determined at autopsy after 10 weeks in sham and TAC-operated cMy-mOVA and cMy-mOVA+OT-I mice. **(B)** Fibrosis of the myocardium was analyzed by Sirius Red staining in cMy-mOVA and cMy-mOVA+OT-I mice after sham and TAC surgery. The bars indicate 100 μm. **(C)** The areas containing collagen were determined on complete left ventricular cross sections of cMy-mOVA (sham *n* = 14, TAC *n* = 10) and cMy-mOVA+OT-I mice (sham *n* = 11, TAC *n* = 9). Means plus SEM are shown in **(A,C)**. The *P*-value of a Kruskal-Wallis test comparing all groups is indicated. Differences between two groups were analyzed by *U*-tests and significant *P*-values are given in the panels (^*^P < 0.05, ^**^P < 0.01, ^***^P < 0.001). The Bonferroni-Holm correction was used to adjust for multiple testing in the two group comparisons.

The cardiac hypertrophy after TAC at autopsy measured as ventricular weight/body weight ratio was similar in cMy-mOVA and cMy-mOVA+OT-I mice (Figure 4A). Myocardial fibrosis was analyzed on Sirius Red stained complete cross-sections (Figures 4A,B) and found to be similarly increased in cMy-mOVA and cMy-mOVA+OT-I mice after TAC (Figures 4A,C).

### OVA-specific CTL can become activated in cMy-mOVA+OT mice after TAC and have a higher cytotoxic activity than OVA-specific CTL in cMy-mOVA mice

**Figure 5:**
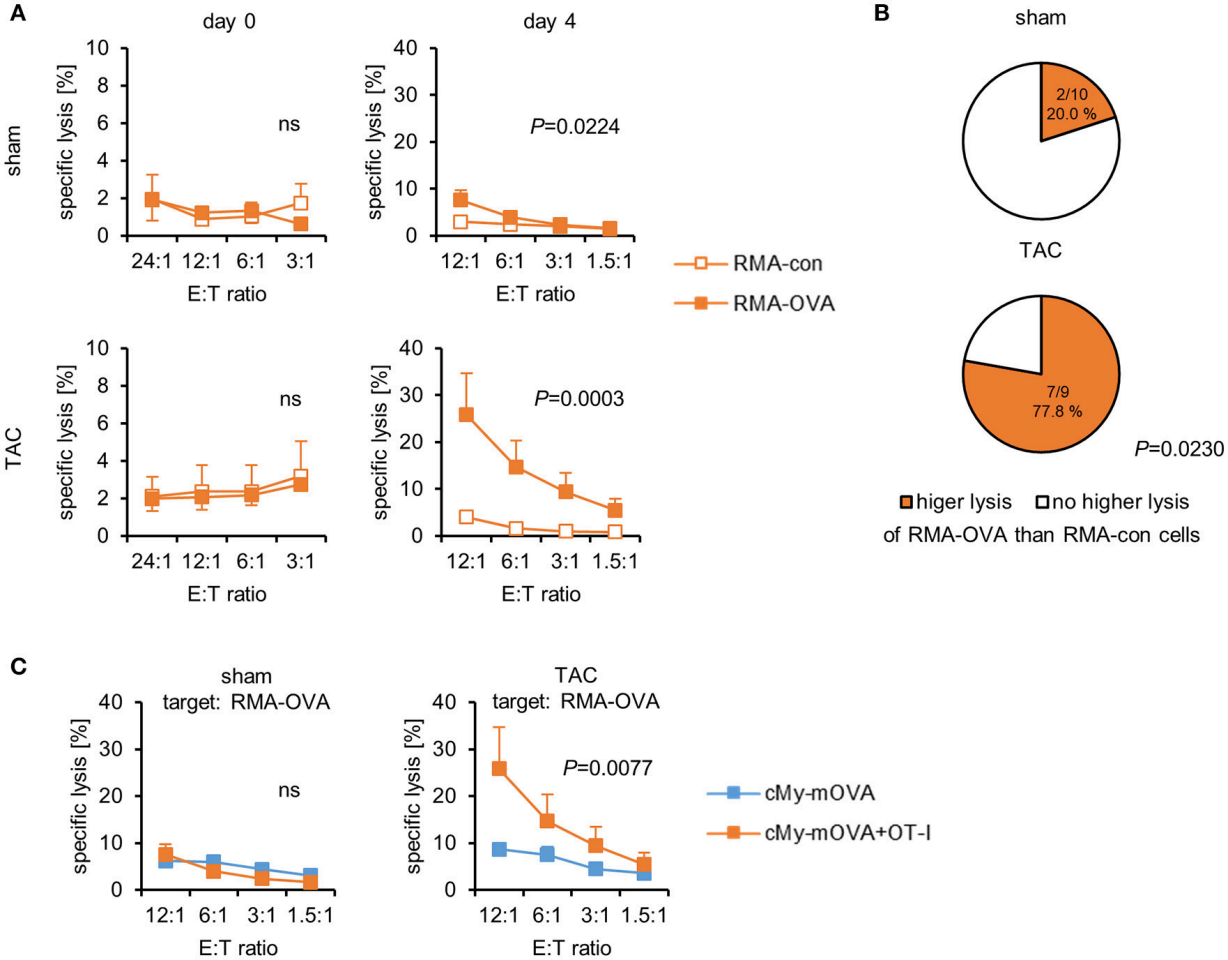
OVA-specific CTL become activated in cMy-mOVA+OT-I mice after TAC and have a higher cytotoxic activity than OVA-specific CTL in cMy-mOVA mice. **(A)** The cytotoxic activity of splenocytes against RMA-OVA and RMA-con target cells was determined by ^51^Cr-release assays at several CD3^+^CD8^+^ E:T ratios 10 weeks after sham (*n* = 10) or TAC (*n* = 9) surgery either directly after autopsy (day 0) or after restimulation *in vitro* with 1 μM OVA (day 4). Means and SEM are displayed. Differences between sham and TAC-operated mice were analyzed by 2-way ANOVA adjusted for the E:T ratios and the respective *P*-values are indicated (ns, non-significant). **(B)** The proportion of mice exerting a higher cytotoxic activity against RMA-OVA than RMA-con cells is displayed. The difference between sham and TAC-operated mice has been analyzed by Fisher‘s exact test and the *P*-value is displayed. **(C)** The OVA-specific cytotoxicity against RMA-OVA target cells of CTL from cMy-mOVA and cMy-mOVA+OT-I mice after sham (*n* = 9) or TAC (*n* = 9) surgery is compared by 2-way ANOVA adjusted for the E:T ratios and the respective *P*-value is indicated.

At autopsy, splenocytes were harvested. The proportion of CD3^+^CD8^+^ cells was analyzed by flow cytometry and found to be similar in mice that had received CD8^+^-T cells from OT-I mice and controls (Supplementary Figure 4E). The proportion of CD3^+^TCRVβ5.1/5.2^+^-T cells was not increased among the splenocytes of cMy-mOVA+OT-I mice (Supplementary Figure 4F), suggesting that the transferred CD8^+^-T cells were either in other compartments or mostly lost at this time point. When the splenocytes were then directly used as effector cells in ^51^Cr-release assays against RMA-con and RMA-OVA target cells, they did not exert any OVA-specific cytotoxicity. However, following a restimulation with 1 μM OVA for 4 days, an OVA-specific cellular cytotoxicity was detected in both sham and TAC-operated mice (Figure 5A). It was much higher in TAC than sham-operated cMy-mOVA+OT-I mice because the RMA-OVA cells were killed significantly better by splenocytes from TAC than sham-operated mice (*P* = 0.009, 2-way ANOVA adjusted for E:T ratios), in contrast to RMA-con cells (*P* = 0.5905, 2-way ANOVA adjusted for E:T ratios). OVA-restimulated splenocytes from a significantly higher proportion of cMy-mOVA+OT-I mice that underwent TAC (77.8%) than sham surgery (20.0%) exerted an OVA-specific cytotoxic activity (*P* = 0.0230, Fisher's exact test; Figure 5B). These frequencies were very similar to those observed in cMy-mOVA mice (Figure 1C). Therefore, we compared the OVA-specific CTL activity in cMy-mOVA and cMy-mOVA+OT-I mice. It was not different in sham-operated cMy-mOVA and cMy-mOVA+OT-I mice but after TAC splenocytes from cMy-mOVA+OT-I mice killed RMA-OVA cells significantly better than splenocytes from cMy-mOVA mice (Figure 5C). In conclusion, this suggests that the risk to elicit OVA-specific CTL activity is not increased after adoptive transfer of OT-I-derived CD8^+^-T cells. However, if (presumably very few) OVA-specific CTL become activated in response to TAC in an animal, they can exert a higher cytotoxic activity upon antigen-specific restimulation, if the high affinity OT-I-derived CD8^+^-T cells had been transferred.

## Discussion

In recent years clear evidence has been accumulated that autoimmune responses can contribute to the progression of heart failure. This is best documented by the presence of autoantibodies with direct cardio-depressive effects in subgroups of patients with dilative cardiomyopathy, in which the therapeutic removal of those antibodies by immunoadsorption is beneficial (11). Notably, similar autoantibodies were also found in animal models of pressure overload (7). More recently, the investigation of the role of T cells in the pathophysiology of pressure overload has been initiated. Our interest in this topic has been stimulated by the finding that pressure overload after TAC, but not volume overload after aorto-caval shunt, was associated with myocardial inflammation and induced a gene expression profile indicating an activation of T cell receptor signaling pathways in the myocardium (16).

The impact of T cells in the pathophysiology of pressure overload has been investigated initially in T cell-deficient and T cell-depleted mice. Mice lacking a functional recombination activation gene 2 (*Rag2*), which do not have B and T cells, were reported to be protected from the transition from hypertrophy to heart failure after TAC (22). Similarly, TCRα-deficient mice or mice in which T cells were depleted by administration of anti-CD3 antibodies, had a preserved cardiac function after TAC (23). Recently, it has been demonstrated that blocking of T cell activation by abatacept, a cytotoxic T-lymphocyte-associated protein 4 (CTLA4)-Ig fusion protein, delays progression of TAC-induced heart failure (24, 25). The question which type of T cells is involved in pressure overload-induced heart failure has also been addressed by some studies. Specifically CD4^+^-T cells appeared to be important since mice deficient for MHC class II molecules, which lack CD4^+^-T cells were protected from progression into heart failure after TAC similarly to RAG2-deficient mice lacking all T cells and in contrast to CD8-deficient mice lacking CD8^+^-T cells (22). We have recently demonstrated that possessing high numbers CD4^+^-T cells with specificity for an antigen in cardiomyocytes can accelerate progression into heart failure after TAC (12). This effect was independent of autoantibodies suggesting that T helper cells can have a direct effect on maladaptive cardiac remodeling.

In the present study, we investigated the role of CD8^+^-T cells with specificity for an antigen in cardiomyocytes during the progression of heart failure in response to pressure overload. We show that CTL with specificity for OVA can become activated after TAC in cMy-mOVA mice expressing OVA in cardiomyocytes. The cytotoxic activity was detected after OVA-specific restimulation of CTL *in vitro*. The CTL activity was on average low, suggesting that its induction is an inefficient process. Moreover, it appeared to be unlikely that such a low-grade CTL activity has a major impact on heart function. This is in agreement with the finding by Laroumanie and colleagues that mice lacking CD8^+^-T cells were not protected from TAC-induced heart failure (22). However, mice possessing higher numbers of CD8^+^-T cells with specificity for antigens in cardiomyocytes might carry a substantially higher risk that such CTL become activated due to pressure overload and subsequently contribute to the deterioration of heart function. To elevate the number of CD8^+^-T cells with specificity for an antigen in cardiomyocytes, we adoptively transferred CD8^+^-T cells with that specificity from OT-I into cMy-mOVA mice before TAC. It has been previously shown that the adoptive transfer of naïve OVA-specific CD8^+^-T cells from OT-I mice into cMy-mOVA mice does not cause myocarditis unless the mice are either immunized with OVA plus a strong adjuvant or are infected with an OVA-expressing virus (13, 26). In agreement with these observations, sham-operated mice that had received OT-I-derived CD8^+^-T cells did not develop any signs of disease in our study. After TAC, these cMy-mOVA+OT-I mice developed a cardiac hypertrophy and progressed into heart failure similarly to cMy-mOVA control mice. Functionally, only the left ventricular dilation was significantly more pronounced in the cMy-mOVA+OT-I than cMy-mOVA mice. Although more T cells infiltrated the myocardium of cMy-mOVA+OT-I than cMy-mOVA mice, the cardiac fibrosis was similar. Systemically, an OVA-specific CTL activity was detectable also in these mice only after *in vitro* restimulation of splenocytes with the antigen. Even though this activity in the cMy-mOVA+OT-I mice was significantly higher after TAC than after sham operation, presumably only very few OVA-specific CTL became activated in response to TAC. This suggests that pressure overload does not provide sufficient danger signals or other adjuvant-like signals to break tolerance and to activate CTL robustly. Although they could exert a higher cytotoxic activity upon antigen-specific restimulation than non TCR-transgenic CTL with that specificity in cMy-mOVA mice, they did not significantly impair cardiac function.

At day 3 after the intervention, transferred OT-I-derived T cells were found in the blood and at one week also in the spleen of TAC and sham-operated mice. At these time points, they constituted 2 to 3% of the peripheral lymphocytes. At the end of the experiment, 10 weeks after the intervention, we did not detect the transferred cells in the spleen by flow cytometry anymore, suggesting that most OT-I-derived OVA-specific T cells were present in other compartments or got lost over time due to absence of an antigenic stimulation. However, the increased OVA-specific cellular cytotoxicity in the spleen and the higher numbers of T cells in heart of cMy-mOVA+OT-I mice indicate that at least some of the transferred T cells got activated after TAC and survived. It should be mentioned that the adoptive transfer even of high numbers of OT-I-derived CD8^+^-T cells (10^7^ in our experiment) into the cMy-mOVA+OT-I mice did not increase the OVA-specific CD8^+^-T cells to similar numbers as reached for OVA-specific CD4^+^-T cells in the double-transgenic cMy-mOVA-OT-II mice, in which stably most CD4^+^-T cells are OVA-specific (12). A constant presence of similar numbers of OVA-specific CD8^+^-T cells could potentially increase the risk to elicit a functionally relevant auto-reactivity also of CTL in cMy-mOVA mice after TAC. Unfortunately, due to the early death of the double-transgenic cMy-mOVA-OT-I mice, this has been impossible to achieve in our experiments. Moreover, it needs to be mentioned that the cMy-mOVA+OT-I displayed a more severe left ventricular dilation 8 weeks after TAC than cMy-mOVA mice. Hence, we cannot exclude that CD8^+^-T cells with specificity for an antigen in cardiomyocytes would impair the cardiac function at later time points beyond the observation period of our study. Transferring OVA-specific CD4^+^-T cells together with OVA-specific CD8^+^-T cells before TAC could potentially support the survival of the OVA-specific CTL and might augment their effects in cMy-mOVA mice.

In comparison to CD4^+^-T cells (12), CD8^+^-T cells with specificity for a model antigen in cardiomyocytes have little impact on the progression of pressure overload-induced heart failure. This observation might be understandable in view of reports that cardiomyocytes largely lack MHC class I molecules under non-inflammatory conditions (18, 27) even though they are inducible by pro-inflammatory cytokines (18) and CTL-mediated killing of cardiomyocytes clearly occurs during viral myocarditis (13). A low expression level of MHC class I molecules on cardiomyocytes could explain why the OVA-specific CD8^+^-T cells had little impact in the cMy-mOVA+OT-I mice after TAC although they reached the OVA-expressing tissue as suggested by the presence of higher numbers of CD3^+^-T cells in the myocardium of these mice. In contrast, in cMy-mOVA-OT-II mice, OVA released from dying cells is expected to be taken-up by professional antigen specific cells, which then stimulate OVA-specific T helper cells to release cytokines involved in cardiac remodeling (12). Therefore, CTL with specificity for antigens in other myocardial cells than cardiomyocytes, e.g., cardiac fibroblasts, might have different consequences for heart function.

## Conclusions

In this study, we have shown that CTL with specificity for a model antigen in cardiomyocytes, i.e., OVA in cardiomyocytes of cMy-mOVA mice, can become activated after TAC. Yet, this apparently is an inefficient process leading only to low-grade cytotoxicity. Adoptive transfer of OVA-specific CD8^+^-T cells from TCR-transgenic OT-I mice does not substantially increase the risk to elicit a cytotoxic activity against OVA after TAC. In agreement with this finding, the progression from cardiac hypertrophy to heart failure was not significantly accelerated in these cMy-mOVA+OT-I mice. Thus, CD8^+^-T cells with specificity for an antigen in cardiomyocytes, in contrast to CD4^+^-T cells (12), apparently do not have a major impact on progression and mortality of pressure overload-induced heart failure.

## Data availability statement

The data that support the findings of this study are available from the corresponding author upon reasonable request.

## Author contributions

RD designed the study. CG, AS, SM, CR, LE, and RD performed experiments and analyzed data. MD and W-HZ supervised echocardiography and the analysis of echocardiography data. VR helped with the adoptive transfers of CTL. GB contributed to the quantitative evaluation of histological data. AL provided the cMy-mOVA mice. KT and GH supervised the mouse surgery. RD wrote the manuscript, which all authors revised. All authors approved the final version of the manuscript.

### Conflict of interest statement

The authors declare that the research was conducted in the absence of any commercial or financial relationships that could be construed as a potential conflict of interest.
