# Supplementary material for: CD8+-T Cells With Specificity for a Model Antigen in Cardiomyocytes Can Become Activated After Transverse Aortic Constriction but Do Not Accelerate Progression to Heart Failure
Source: Front Immunol. 2018 Nov 15;9:2665. doi: 10.3389/fimmu.2018.02665 (PMC6249381; doi:10.3389/fimmu.2018.02665)
Supplement: Supplementary file 1 [file Data_Sheet_1.PDF]

Supplementary Material

CD8$^+$-T cells with specificity for an antigen in cardiomyocytes can become activated after transverse aortic constriction but do not accelerate progression into heart failure

Carina Gröschel, André Sasse, Sebastian Monecke, Charlotte Röhrborn, Leslie Elsner, Michael Didié, Verena Reupke, Gertrude Bunt, Andrew H. Lichtman, Karl Toischer, Wolfram-Hubertus Zimmermann, Gerd Hasenfuß, Ralf Dressel*

*** Correspondence:** Corresponding Author: rdresse@gwdg.de

Supplementary Figures

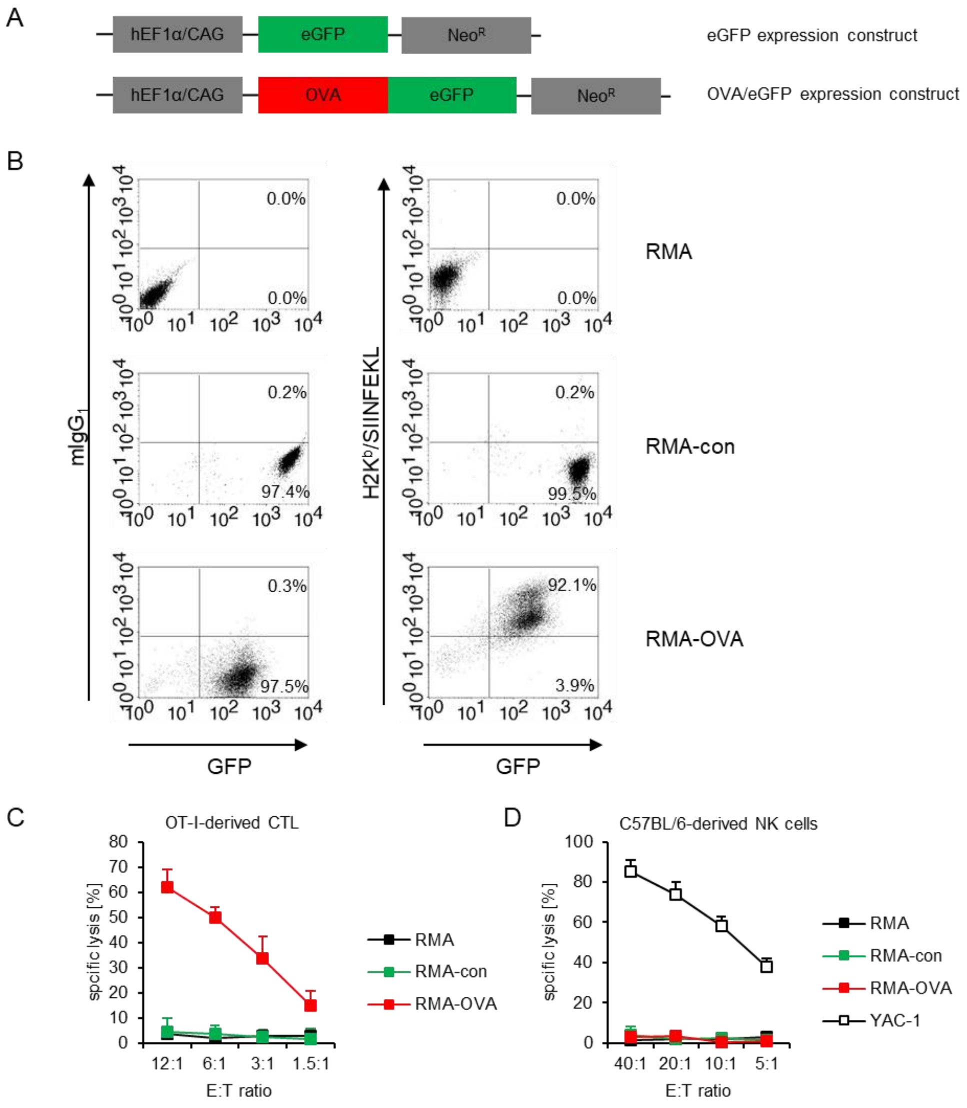

Supplementary Figure 1. RMA-OVA cells express OVA and are killed by OVA-specific CTL from OT-I mice. (**A**) The constructs for expression of eGFP or an OVA/eGFP fusion protein under a hEF1α/CAG promoter are schematically represented. The constructs contain a neomycin resistance gene to allow for selection of stable transfectants. (**B**) Parental RMA cells, the stable clone of RMA-con cells transfected with the eGFP expression construct and the stable clone transfected with the OVA/GFP expression construct used in subsequent experiments were analyzed by flow cytometry for the expression of GFP and the OVA-derived peptide SIINFEKL presented by $H2K^b$ molecules. A mouse IgG_1 isotype control was used for the $H2K^b$/SIINFEKL-specific monoclonal antibody. (**C**) The cell lines were also used as targets for OT-I-derived CTL, which recognize the peptide SIINFEKL in an $H2K^b$-restricted manner. The OVA-dependent killing of the target cells was determined by ^{51}Cr-release assays at several $CD3^+CD8^+$ effector to target (E:T) ratios after *in vitro* restimulation of splenocytes from OT-I mice with 1 µM OVA for 4 days. (**D**) NK cells failed to kill the parental RMA cells and the transfected clones. NK cells were purified by MACS from spleens of C57BL/6 mice before being used in ^{51}Cr-release assays. YAC-1 target cells served as positive control. Means + SEM of triplets are shown in panels **C** and **D** for a representative experiment.

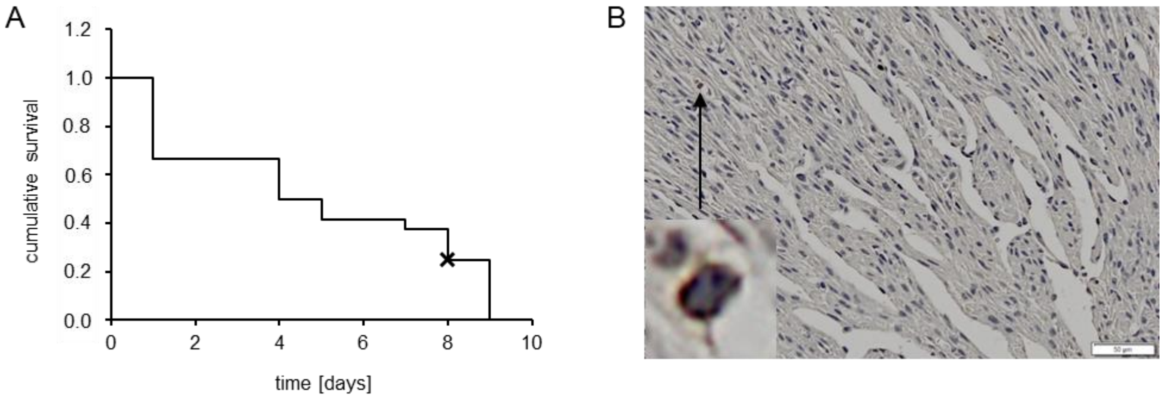

Supplementary Figure 2. The cMy-mOVA-OT-I are not viable. (**A**) The Kaplan-Meier survival curve of 24 cMy-mOVA-OT-I mice from three litters is shown. At day 8, five mice were sacrificed for histological analyses. (**B**) The presence of $CD3^+$-T cells in the myocardium of the cMy-mOVA-OT-I mice was determined by immunohistochemistry and found to be very low as illustrated here exemplarily. One $CD3^+$-T cell was found in the displayed area, which is shown enlarged in the inlet. The bar indicates 50 µm.

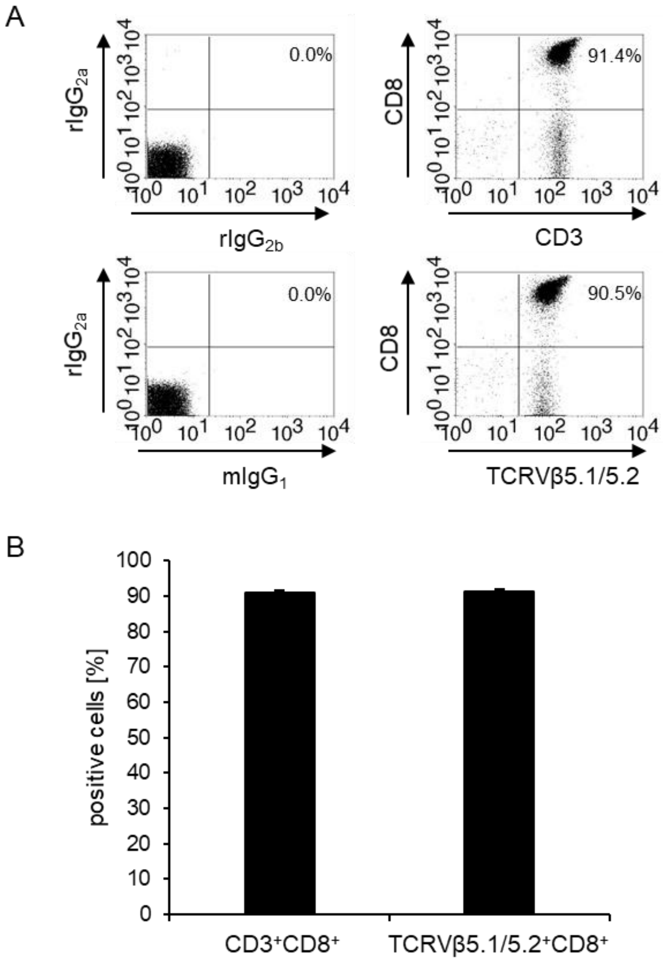

Supplementary Figure 3. Separation of $CD8^+$-T cells from OT-I mice for adoptive transfer. (A) Lymphocytes were obtained from lymph nodes of OT-I mice and $CD8^+$-T cells were separated by MACS. The purity of the cell suspension was analyzed by flow cytometry as illustrated here. Always more than 90 % of the T cells were $CD8^+$ cells expressing the transgenic TCR, which is stained by an anti-TCRVβ5.1/5.2 antibody. (B) Means + SEM of three cell populations obtained by MACS and used in adoptive transfer experiments are shown.

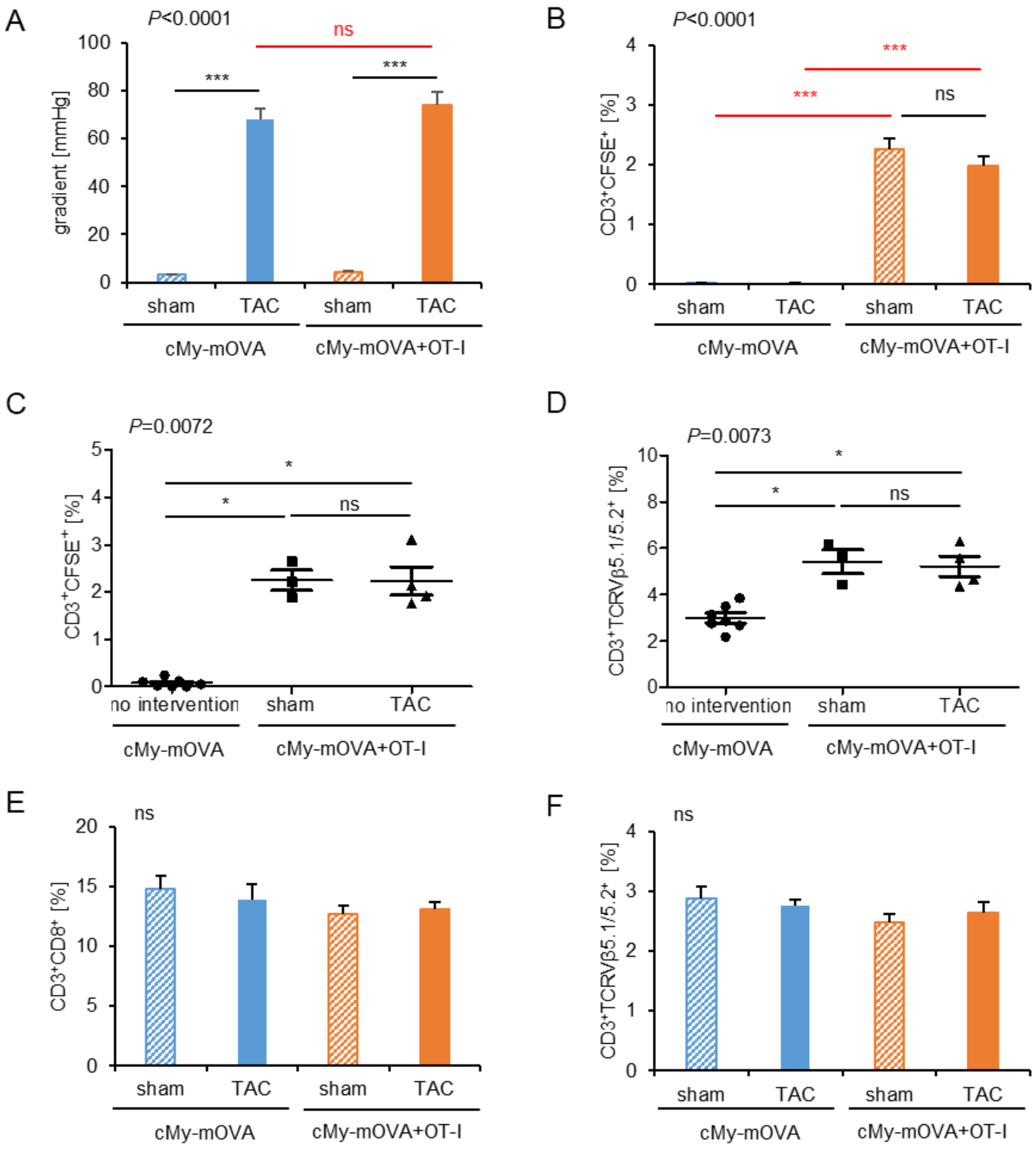

Supplementary Figure 4: Aortic stenosis after TAC in cMy-mOVA mice and presence of OT-I-derived T cells in cMy-mOVA+OT-I mice after transfer. (**A**) The pressure gradient over the aortic ligature was determined in the sham and TAC-operated mice using pulsed wave Doppler. Means + SEM are shown for cMy-mOVA (n=14 per group) and cMy-mOVA+OT-I mice (n=11 per group). The *P*-value of a Kruskal-Wallis test comparing all groups is indicated. U-tests comparing sham and TAC-operated mice are indicated in black and *P*-values comparing cMy-mOVA and cMy-mOVA+OT-I mice in red font. The Bonferroni-Holm correction was used to adjust for multiple testing in the two group comparisons (****P*<0.001; ns: non-significant). (**B**) In parallel at day 3 after sugery, the proportion of $CD3^{+}CFSE^{+}$ OT-I-derived cells among blood lymphocytes was determined by flow cytometry. Means + SEM are shown and the *P*-value of a Kruskal-Wallis test comparing all groups is indicated. U-tests comparing sham and TAC-operated mice are indicated in black and *P*-

values comparing cMy-mOVA and cMy-mOVA+OT-I mice in red font. The Bonferroni-Holm correction was used to adjust for multiple testing in the two group comparisons. (**C**) Additional cMy-mOVA+OT-I mice (sham: n=3, TAC: n=4) were operated and sacrificed one week after the intervention together with cMy-mOVA mice that were not operated (n=7). The proportion of $CD3^{+}CFSE^{+}$ OT-I-derived cells among splenocytes was determined by flow cytometry. Means ± SEM are shown and the *P*-value of a Kruskal-Wallis test comparing all groups is given. Results of U-tests comparing two groups and adjusted for multiple testing by the Bonferroni-Holm correction are indicated (**P*<0.05). (**D**) In parallel, the proportion of $CD3^{+}TCRV\beta5.1/5.2^{+}$-T cells among splenocytes of these mice was determined by flow cytometry and analysed in the same way. (**E**) The proportion of $CD3^{+}CD8^{+}$ cells among splenocytes of sham and TAC-operated cMy-mOVA (sham n=14, TAC n=10) and cMy-mOVA+OT-I mice (sham n=11, TAC n=9) is shown as determined by flow cytometry at autopsy 10 weeks after the intervention. Means + SEM are shown and the result of a Kruskal-Wallis test comparing all groups is indicated. (**F**) In parallel, the proportion of $CD3^{+}TCRV\beta5.1/5.2^{+}$-T cells was measured and analysed in the same way.

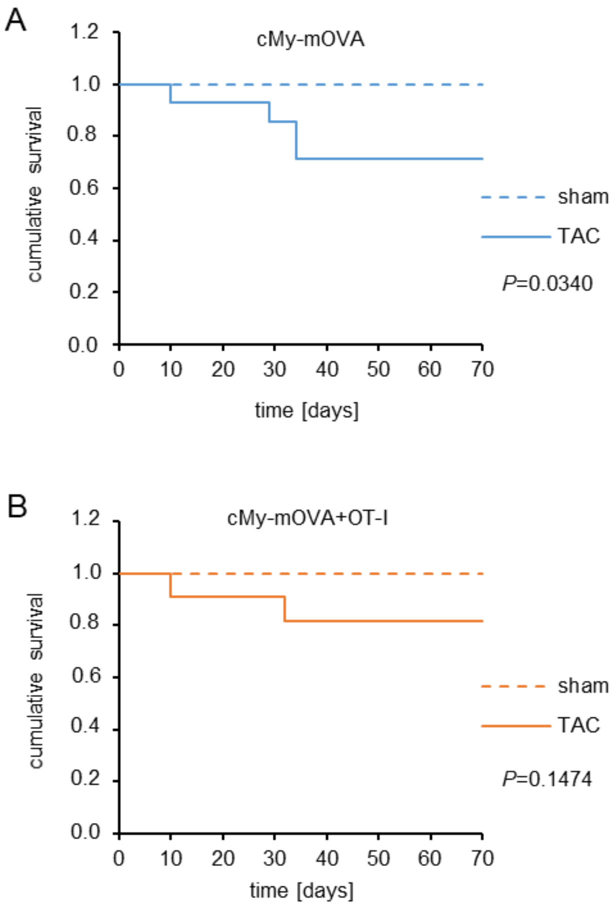

Supplementary Figure 5: Survival of sham and TAC-operated cMy-mOVA and cMy-mOVA+OT-I mice. Kaplan-Meier survival curves for sham and TAC-operated (**A**) cMy-mOVA and (**B**) cMy-mOVA+OT-I mice are displayed. The survival of sham and TAC-operated mice was compared by Log-rank tests (Cox-Mantel) and the respective *P*-values are given.
